# Effect of the COVID-19 pandemic on patients with inherited neuromuscular disorders

**DOI:** 10.1590/0004-282X-ANP-2021-0166

**Published:** 2022-08-08

**Authors:** Cristiane Araujo Martins Moreno, Clara Gontijo Camelo, Pedro Henrique Marte de Arruda Sampaio, Alulin Tácio Quadros Santos Monteiro Fonseca, Eduardo de Paula Estephan, André Macedo Serafim Silva, Renann Nunes Pirola, Luiz Henrique Libardi Silva, Karlla Danielle Ferreira Lima, Marco Antônio Veloso de Albuquerque, Antonio Edvan Camelo, Marcos Vinícius Oliveira Marques, Mario Teruo Yanagiura, Wagner Cid Palmeira Cavalcante, Ciro Matsui, Lucas Michielon de Augusto Isihi, Rodrigo Holanda Mendonça, Ana Flávia Pincerno Pouza, Mary Souza de Carvalho, Umbertina Conti Reed, Edmar Zanoteli

**Affiliations:** 1 Universidade de São Paulo, Faculdade de Medicina, Departamento de Neurologia, São Paulo SP, Brazil.

**Keywords:** Neuromuscular Diseases, COVID-19, Social Isolation, Consequence Analysis, Doenças Neuromusculares, COVID-19, Isolamento Social, Análise de Consequências

## Abstract

**Background:**

The COVID-19 pandemic has brought substantial challenges for current practices in treating hereditary neuromuscular disorders (hNMDs). However, this infection has not been the only concern for these patients. Social distancing has compromised multidisciplinary assistance and physical activity, and has brought about several mental health issues. We presented a follow-up on 363 patients with hNMDs at a Brazilian tertiary center during the peak of the COVID-19 pandemic.

**Objective:**

We aimed to show the frequency and severity of SARS-CoV-2 infection among hNMD patients and to demonstrate the effects of the pandemic on life habits, disease progression and multidisciplinary supportive care status.

**Methods:**

Three hundred and sixty-three patients (58% male and 42% female) were followed for three months through three teleconsultations during the peak of the COVID-19 pandemic in Brazil.

**Results:**

There were decreases in the numbers of patients who underwent physical, respiratory and speech therapies. For several patients, their appetite (33%) and sleep habits (25%) changed. Physical exercises and therapies were interrupted for most of the patients. They reported new onset/worsening of fatigue (17%), pain (17%), contractions (14%) and scoliosis (7%). Irritability and sleep, weight and appetite changes, and especially diminished appetite and weight loss, were more frequent in the group that reported disease worsening. There was a low COVID-19 contamination rate (0.8%), and all infected patients had a mild presentation.

**Conclusion:**

The isolation by itself was protective from a COVID-19 infection perspective. However, this isolation might also trigger a complex scenario with life habit changes that are associated with an unfavorable course for the NMD.

## RESUMO

**Antecedentes:**

A Pandemia por COVID-19 tem trazido desafios subtanciais para a prática clínica no tratamento das doenças neuromusculares hereditárias (DNMh). A infecção não tem sido a única preocupação para os pacientes. O distanciamento social tem comprometido a assistência multidisciplinar, atividade física e tem trazido problemas mentais em decorrência do próprio isolamento. Nós apresentamos aqui um seguimento de 363 pacientes com DNMh de um centro terciário Brasileiro durante o pico da Pandemia de Covid-19. Objetivos: Mostrar a frequência e gravidade da infecção por Sars-Cov-2 em pacientes com DNMh e demonstrar os efeitos da pandemia nos hábitos de vida, na progressão da doença e no cuidado multidisciplinary.

**Métodos:**

Trezentos e sessenta e três pacientes (58% homens and 42% mulheres) foram acompanhados por 3 meses através de 3 teleconsultas durante o pico da Pandemia de Covid-19 no Brasil.

**Resultados:**

Houve um decréscimo no número de pacientes que faziam terapia física, respiratória e fonoaudiológica. Em muitos pacientes, o apetite (33%) e hábitos do sono (25%) se alteraram. Exercícios físicos e terapias foram interrompidas pela maioria dos pacientes. Physical exercises and therapies were interrupted for most of the patients. Eles relataram piora ou aparecimento de fadiga (17%), dor (17%), retrações (14%), e escoliose (7%). Irritabilidade, mudanças no sono, peso e apetite, sendo principalmente diminuição do apetite e peso foram mais frequentemente encontrados em pacientes que apresentaram piora clinica da doença. Houve uma baixa taxa de contaminação por Covid-19 (0.8%), e todos os pacientes infectado apresentaram quadro clinico leve.

**Conclusão:**

O isolamento por si só se mostrou protetor na perspectiva de infecção por Covid-19, mas pode desencadear um cenário complexo com mudanças nos hábitos de vida e curso desfavorável da doença de base.

## INTRODUCTION

The COVID-19 pandemic has been significantly changing current practices for treating neurological disorders and has brought substantial challenges among the different subgroups of disorders^1^ ^-^ ^6^. Inherited neuromuscular diseases (hNMDs) constitute a group of heterogeneous conditions affecting both children and adults to a degree that varies widely from one patient to another. They include diseases affecting muscles, neuromuscular junctions, peripheral nerves, metabolic systems and motor neurons. A significant number of these patients display great disability, and many of them present cardiac, respiratory and/or bulbar involvement in addition to muscle weakness^7^ ^,^ ^8^. 

COVID-19 has brought up several issues for the field of neuromuscular disorders. Many specialists in this field have adopted a state of vigilance due to the potential neuromuscular complications of SARS-CoV-2 infection^9^ ^,^ ^10^. However, there has not been much discussion about how to assess the risk of severe COVID-19 presentations among neuromuscular patients. It has been speculated that heightened risk would be attributable to the presence of comorbidities, older age and use of immunotherapies that patients might be receiving. Moreover, patients without swallowing and breathing difficulties would not be considered to be in the high-risk category^1^.

In this pandemic scenario, the infection itself is not the only concern for neuromuscular patients. Although social distancing was established as the only effective measure for diminishing the risk of contamination, the isolation by itself has compromised medical assistance and multidisciplinary supportive therapies and limited physical activity. These support measures are directly involved in enabling a better long-term prognosis for hNMDs.

In Brazil, the epidemic phase of COVID-19 began in March 2020 and resulted in the implementation of emergency measures and cancelation of routine neurological appointments at our reference center. The neuromuscular clinic promptly switched its in-person appointments to teleconsultations. The main goals were to protect the patients from contamination and to ensure continuity of their treatment. The follow-up allowed us to collect the patients’ perceptions of their disease progression and any comorbidities that they may have presented during this time. 

In this study, we present the follow-up of 363 patients with hNMDs who were being attended through a Brazilian tertiary center during the peak of the COVID-19 pandemic. We aimed to show the frequency and severity of SARS-CoV-2 infection in this group of patients and to demonstrate the effects of the pandemic and the consequent restrictive measures regarding psychological factors, disease progression and multidisciplinary supportive care status for the patients.

## METHODS

We reviewed the clinical database of the neuromuscular clinic of HC/FMUSP and we were able to select 468 eligible patients with clinically, histologically and/or genetically confirmed hNMD whose last appointment at the neuromuscular disorders section of Hospital das Clínicas, São Paulo, Brazil, was more recent than 2016. Phone calls were made by the authors every 30 days up to a total of three calls per patient between April and July 2020. Adult patients were required to answer for themselves, while for patients below the age of 18, parents or a legal guardian were required to respond. 

All information was collected using a questionnaire that was designed for the NMD teleconsultations during the pandemic period. The first part of the questionnaire asked for pre-pandemic data regarding the following: on maximum motor skill; need for/use of respiratory support and feeding devices; presence of tracheostomy; use of medications for treatment of the NMD condition; regular practice of physical (PT), respiratory (RT) and speech therapy (ST); presence of comorbidities (cardiopathy, obesity, diabetes and asthma); and whether the patient was in a home care regime. The second part covered the pandemic period and asked about (1) access to therapies; (2) psychological factors, socioeconomic factors aspects and life habits (irritability, fear, appetite changes [yes, more or less], sleep changes [yes, more or less], weight changes [yes, gain or loss] and the patient’s family income [stable, more or less]); (3) COVID-19 infection (whether the patient or a close family member had presented signs and symptoms of confirmed/suspected COVID-19 infection); and (4) the patient’s and/or family member’s perception of disease progression and new onset or worsening of preexisting symptoms related to the hNMD during the pandemic period (fatigue, pain, independence in daily life activities, duration of respiratory support use, swallowing difficulty, cardiopathy, contractures and scoliosis).

The study protocol was approved by the local IRB. Statistical analysis was carried out by means of the R software. A chi-square test for categorical variables was performed using the Yates correction, and Fisher’s exact test was used to check differences between the group of patients (both bed-restricted and walking patients) who reported disease worsening and the group that said that their own condition was stable. 

## RESULTS

Data were collected from the medical records of 468 patients. These patients were followed for three months through three teleconsultations. Subjects who did not undergo all three phone-call assessments were excluded from the final analysis. This resulted in retention of 363 patients in this analysis (78%); of these, 58% were male and 42% female. 

The patients had the following diagnoses: congenital myopathy (CM), congenital muscle dystrophy (CMD), congenital myasthenic syndrome (CMS), spinal muscle atrophy (SMA), facioscapulohumeral dystrophy (FSH), limb girdle muscle dystrophy (LGMD), hereditary neuropathy (Charcot-Marie-Tooth [CMT]), Duchenne and Becker muscular dystrophies (DMD/BMD), mitochondrial disease (MitoD), Pompe disease (PD) and myotonic dystrophy type 1 (DM1). The findings were divided according to the disease group between pre-pandemic findings (Table 1) and post-pandemic findings (Table 2).

**Table 1.** Self-report findings from 363 patients with hNMD during the COVID-19 pandemic, over a three-month follow-up period: pre-pandemic data.

		CM/CMS	CMD	ColVI	MD1	BMD	DMD	FSH	LGMD	MitoD	CMT	Pompe	SMA_I	SMA_II/III	Total
Demographics	Patients (count)	63	36	8	34	19	31	10	34	42	26	8	11	41	363
	Age (years)	21 (± 12)	10 (± 5)	14 (± 3)	44 (± 15)	33 (± 14)	17 (± 15)	51 (± 15)	38 (± 26)	41 (± 21)	19 (± 9)	46 (± 6)	5 (± 3)	11 (± 6)	26 (± 20)
	Sex (male/female)	32/31	16/20	6/2	22/12	17/2	31/0	7/3	16/18	19/23	13/13	4/4	4/7	25/16	212/151
Baseline	Cardiopathy	1	-	-	11	7	14	2	4	7	2	-	-	-	48 (13.2%)
	Asthma	4	5	2	1	-	-	-	3	-	7	-	-	1	23 (6.3%)
	Diabetes	1	-	-	4	1	-	3	1	5	-	-	-	-	15 (4.1%)
	Overweight	7	1	-	8	5	4	2	12	2	1	2	-	4	48 (13.2%)
	Flu vaccine	51	27	7	28	16	23	7	25	36	17	6	8	31	282 (77.7%)
	Continuous med. intake	19	12	5	19	6	15	4	13	18	10	4	6	17	148 (40.8%)
Disease burden	Bed-restricted	1	-	-	-	-	-	-	-	-	2	-	7	-	10 (2.8%)
	Few movements	1	-	-	-	-	-	-	1	-	-	-	2	4	8 (2.2%)
	Roll over	2	-	-	-	-	-	-	1	-	2	-	9	4	18 (5.0%)
	Wheelchair-bound	-	4	-	-	-	-	-	3	1	1	-	-	11	20 (5.5%)
	Sit with support	6	22	2	2	1	12	-	5	1	2	-	-	16	69 (19.0%)
	Sit without support	5	5	-	-	-	-	-	2	1	1	-	2	1	17 (4.7%)
	Stand with support	5	5	-	-	-	-	-	2	1	1	-	2	1	17 (4.7%)
	Stand with support	16	36	2	2	1	12	-	12	4	5	-	4	29	123 (33.9%)
	Walk	50	5	6	32	18	19	10	23	39	20	8	-	9	239 (65.8%)
	A few steps	1	-	1	-	2	-	-	1	-	1	-	-	2	8 (2.2%)
	Bilateral support	3	-	-	3	-	2	-	-	-	1	1	-	-	10 (2.8%)
	Unilateral support	3	-	-	7	1	-	3	8	3	3	2	-	1	31 (8.5%)
	Short distances	5	1	2	9	6	5	3	6	1	1	-	-	1	40 (11.0%)
	Medium/long distance	38	4	3	13	9	12	4	8	35	14	5	-	5	150 (41.3%)
	TQ	5	-	-	-	-	-	-	-	1	1	-	8	3	18 (5.0%)
	VS continuous	3	-	-	-	-	-	-	-	1	1	-	8	4	17 (4.7%)
	VS intermittent	5	2	-	3	-	-	-	1	1	-	1	1	4	18 (5.0%)
	VS nighttime	14	15	1	10	1	-	1	3	1	-	1	1	17	65 (17.9%)
	No VS	41	19	7	21	18	31	9	30	39	25	6	1	16	272 (74.9%)
	GTT only	4	1	-	-	1	-	-	-	1	2	-	8	3	20 (5.5%)
	GTT/mouth tasting	3	1	-	-	-	-	-	-	1	-	-	1	-	6 (1.7%)
	GTT/mouth	1	1	-	-	-	-	-	-	-	-	-	-	3	5 (1.4%)
	Mouth mashed food	5	21	1	7	-	1	1	2	9	1	1	-	7	59 (16.3%)
	Mouth only	50	12	7	27	18	30	9	32	31	23	7	2	28	282 (77.7%)

CM: congenital myopathy; ADG: alpha-dystroglycanopathy; ColVI: collagen VI myopathy; CMS: congenital myasthenic syndrome; MD1: myotonic dystrophy type 1; BMD: Becker muscle dystrophy; DMD: Duchenne muscle dystrophy; FSH: facioscapulohumeral dystrophy; LGMD: limb girdle muscle dystrophy; MitoD: mitochondrial disorders; CMT: Charcot-Marie-Tooth; Pompe: Pompe disease; SMA: spinal muscular atrophy; M: male; F: female; Med: medication; TQ: tracheostomy; VS ventilatory support equipment (bipap); GTT: gastrostomy tube.

**Table 2.** Data collected after four months of isolation due to COVID-19 pandemic: pandemic data.

		CM/CMS	CMD	ColVI	DM1	DMB	DMD	FSH	LGMD	MitoD	CMT	Pompe	SMA_I	SMA_II/III	Total
During pandemic	Difficulty in obtaining medication	7	4	1	3	1	6	1	-	3	3	1	-	5	35 (9.6%)
	Fear (n = 314)*	19	15	1	17	4	11	3	17	15	13	4	-	14	135 (43.3%)
	Irritability (n = 314)*	22	8	1	13	5	15	2	15	8	11	3	-	15	118 (37.6%)
	Sleeping more	10	8	-	6	4	6	-	8	-	3	-	1	3	49 (13.5%)
	Sleeping less	6	7	-	7	1	5	-	4	3	2	1	1	4	41 (11.3%)
	Eating more	14	12	3	10	2	9	2	14	8	8	2	3	10	97 (26.7%)
	Eating less	3	5	-	3	1	5	1	5	2	1	-	-	2	28 (7.7%)
	Gained weight	22	18	2	13	2	11	2	16	11	7	1	2	7	114 (31.4%)
	Lost weight	3	2	-	2	3	5	1	5	4	2	-	-	3	30 (8.3%)
Therapies	PT_PrePandemic	31	31	7	10	12	26	7	19	21	17	6	11	39	237 (65.3%)
	PT_Pandemic	9	6	4	17	4	8	2	11	11	7	1	1	5	86 (23.7%)
	PT_Pandemic_alternative	18	9	-	5	1	8	1	6	7	4	1	3	10	73 (20.1%)
	RT_PrePandemic	16	21	4	3	1	9	1	8	10	2	3	11	30	116 (32.0%)
	RT_Pandemic	5	7	3	10	3	6	-	8	7	2	-	-	1	48 (13.2%)
	RT_Pandemic_alternative	6	7	-	2	-	6	-	1	1	1	-	1	3	23 (6.3%)
	ST_PrePandemic	11	22	1	-	-	-	1	3	10	1	2	9	22	78 (21.5%)
	ST_Pandemic	4	11	2	5	-	2	-	4	6	2	-	-	3	32 (8.8%)
	ST_Pandemic_alternative	3	8	-	1	1	-	-	-	-	-	-	1	-	6 (1.7%)
COVID-19 infection	Close_contact_suspected	11	7	4	8	2	4	-	7	6	5	-	3	15	72 (19.8%)
	Not_tested	8	4	4	6	1	3	-	6	2	3	-	1	5	43 (11.8%)
	Tested (-)	0	1	-	1	-	-	-	1	3	1	-	-	-	7 (1.9%)
	Tested (+)	3	2	-	1	1	1	-	-	1	1	-	2	10	22 (6.1%)
	Patient_suspected	7	5	-	8	2	-	1	3	4	1	1	3	3	38 (10.5%)
	Not_tested	6	4	-	6	-	-	1	1	4	-	-	2	2	26 (7.2%)
	Tested (-)	1	-	-	2	2	-	-	2	-	1	1	-	-	9 (2.5%)
	Tested (+)	0	1	-	-	-	-	-	-	-	-	-	1	1	3 (0.8%)
Self-perception of disease progression after four months of the pandemic	Fatigue	6	5	-	12	2	7	-	10	6	5	-	-	8	61 (17%)
	Pain	7	5	1	7	2	4	-	11	11	4	-	1	6	62 (17%)
	More dependent (DLA)	9	5	-	9	-	9	2	5	4	2	-	1	6	55 (15%)
	Respiratory dysfunction	1	-	-	1	-	1	-	1	-	-	-	-	2	7 (2%)
	Swallowing dysfunction	1	-	-	1	1	1	-	1	-	-	-	-	-	5 (1.3%)
	Cardiopathy	0	-	-	-	-	-	-	1	1	-	-	-	-	3 (0.8%)
	Contractures	3	8	-	7	-	6	2	11	4	5	-	1	5	54 (14.5%)
	Scoliosis	1	1	-	4	-	2	-	6	4	5	-	1	-	27 (7.3%)
	General	10	12	1	14	3	11	4	15	6	6	-	2	8	97 (26.1%)

*Data referring to fear and irritability were included only for patients older than 7 years of age.

CM: congenital myopathy; ADG: alpha-dystroglycanopathy; ColVI: collagen VI myopathy; CMS: congenital myasthenic syndrome; MD1: myotonic dystrophy type 1; BMD: Becker muscle dystrophy; DMD: Duchenne muscle dystrophy; FSH: facioscapulohumeral dystrophy; LGMD: limb girdle muscle dystrophy; MitoD: mitochondrial disorders; CMT: Charcot-Marie-Tooth; Pompe: Pompe disease; SMA: spinal muscular atrophy; PT: physical therapy; RT: respiratory therapy; ST: speech therapy; DLA: daily life activities.

### Baseline clinical characteristics of the patients

Baseline information was collected from the patients’ charts and was confirmed in the first teleconsultation, which focused on the pre-pandemic period. The patients’ average age was 26 (± 20) years; 42% were male, 66% were able to walk, 38% were wheelchair-bound and 5% were bed-restricted. Most of the patients (93%) were orally fed; 74% did not need ventilatory support (VS), 17% needed it only during the night and 9% needed it intermittently or continuously. Tracheostomy was present in 4% of the patients. Cardiopathy was referred to by 13% of the patients, asthma by 7%, diabetes by 4% and obesity by 13%.

### Rehabilitation during the pandemic period

There were decreases in the numbers of patients who underwent in-person PT (p < 0.001), RT (p < 0.001) and ST (p < 0.001) during the pandemic period. However, some of the patients looked for alternatives such as doing exercises with family members or online with their therapists.

### COVID-19 infection

COVID-19 infection was suspected in 38 patients. Among these, three were admitted to a hospital, stayed for fewer than seven days and tested negative for SARS-CoV-2, whereas 35 had only mild symptoms that did not require hospitalization. Tests were performed only on nine patients, among which three were positive. Symptomatic close family members were reported in the cases of 72 patients, including 22 who tested positive for COVID-19, but the patients had no symptoms within 30 days after the contact.

### Patients’ reports during the pandemic period

Sleep pattern changes were reported by 25% of the patients, appetite changes by 33% and weight changes by 39%. Concerning new onset or worsening of the preexisting symptoms related to the hNMD, 17% of the patients reported fatigue, 17% reported pain, 15% complained that they were more dependent for daily activities, 14.5% noticed that their contractures were worse and 7.3% considered that their scoliosis was aggravated. In addition, 2% of the patients indicated that they needed more time on VS, 1.3% experienced more swallowing issues and 0.8% reported cardiopathy worsening.

At the last evaluation, disease worsening was noticed by 6% of the bed-restricted patients, 27% of the wheelchair-bound patients and 27% of the patients who were able to walk. Among the disease groups, patients with LGMD were the ones who complained most of disease worsening. In total, 44% of them reported worsening, followed by patients with DM1 (40%), DMD (40%), FSH (40%) and CMD (30%). However, there was no statistical difference between them. A comparison was made between the group of patients (both bed-restricted and walking patients) who reported disease worsening (n = 84) and the group who reported that they were stable (n = 240). There was no statistical difference in average age with regard to motor, respiratory and bulbar baseline status between the two groups. Patients who complained about worsening of their disease also reported more irritability (p = 0.003; OR = 2), sleep changes (p < 0.001; OR = 2.78), weight changes (p < 0.001; OR = 2.77), weight loss (p = 0.002; OR = 3.47), appetite changes (p < 0.001; OR = 2.48) and low appetite (p < 0.001; OR = 4.16). 

## DISCUSSION

 Social isolation and cancelations of in-person consultations were shown to decrease contamination rates but brought about several issues for this population, which continuously requires physical exercise, PT, RT and ST, as well as multidisciplinary medical appointments.

There were not enough data to conclusively determine the severity of COVID-19 among the hNMD patients in this cohort. However, all the suspected patients had mild symptoms. Only three suspected patients (1 SMA_I, 1 SMA_II and 1 LAMA2) tested positive. In spite of their severe hNMD disease, all of them had mild COVID-19 symptoms. An additional 20% of the patients had close contact with a suspected COVID-19 patient, but they had no related symptoms either prior to or after the contact. We speculate that our low infection rates and low severity of cases were due to the low rates of comorbidities such as obesity and diabetes, as well as the low average age. Previous data had shown that pediatric COVID-19 cases tended to be less severe than adult cases and that children were less likely to become positive when exposed^11^. Patients receiving immunosuppressive treatment were expected to have worse outcomes^1^ ^,^ ^10^. In our cohort, only the DMD patients were using corticosteroids, but none of them were suspected or confirmed to have COVID-19. There was no information about asymptomatic carriers because we were unable to perform extensive COVID-19 testing. 

Di Stefano et al (2020) demonstrated that there was decreased physical activity among hNMD patients during the pandemic period and showed that less exercise correlated directly with diminished quality of life^12^. Handberg et al (2021) also reported that health and physical functioning decreased and changes to access to physiotherapy or healthcare occurred due to the pandemic, thus demonstrating that the pandemic had had a negative effect on the biopsychosocial health and quality of life of patients with neuromuscular diseases^13^. Our data agree with this finding. We found that an impressive number of patients had reduced their in-person PT, RT, and ST, going from 65%, 31% and 21% prior to the pandemic period, down to 23%, 13% and 9%, respectively. This information indicates that more than half of the patients who regularly underwent rehabilitation therapies before the pandemic period interrupted them for at least four months. 

We found that 27% of the patients complained of disease worsening after four months of social isolation. These patients were distributed within all the diagnostic groups except PD, CMS and MitoD. As expected, worsening of symptoms occurred less frequently among bed-restricted patients, among whom only 6% had any complaint, versus 27% of the wheelchair-bound patients and 27% of the patients who were able to walk. Most of the bed-restricted patients were in a home care regime and were less affected by the pandemic restrictions. We speculate that self-assessment would not be able to capture small changes in such severely affected patients.

The main complaints relating to disease progression were worsening of the contractures, fatigue and pain. Patients reported that they were becoming more dependent for daily life activities. There was no statistically significant difference between patients who continued their therapies and those who discontinued. We compared the group of patients (both bed-restricted and walking patients) who reported disease worsening with the group that reported that their condition was stable. We noticed that average age, comorbidities and severity of the disease at the baseline assessment were not correlated with the perception of disease worsening. However, irritability and sleep, weight and appetite changes, and especially diminished appetite and weight loss, occurred more frequently in the group that reported disease worsening. It is not possible to infer whether these symptoms might be the cause of the worsening or whether they are only red flags for a complex pandemic scenario involving mental health issues. The psychosocial effects of the pandemic are a significant concern, and patients considered to be at heightened risk of severe COVID-19 presentations, such as hNMD patients, are more vulnerable to such effects^14^. 

This study had several limitations. The data were collected using phone calls and were dependent on the patients’ and/or parents’ perceptions of their disease. An in-person follow-up would have been ideal for evaluating how the patients were really affected by the pandemic, in addition to enabling a detailed psychological evaluation.

In conclusion, the isolation might have been protective from the perspective of COVID-19 infection, but for hNMD patients, it brought on new symptoms and/or aggravated previous ones such as pain, joint retraction and fatigue. Reduction of physical exercises and therapies may have had a catastrophic impact on daily life activities and disease progression during the pandemic period. However, sleep, weight and appetite changes may also have played an important role in the disease and these need to be routinely evaluated for hNMD patients. We emphasize the importance of keeping social distancing and maintaining all healthcare measures in order to avoid contamination. However, it is also important to encourage hNMD patients to keep doing physical exercises and to keep track of their eating and sleeping habits, so as to avoid disease complications and an unfavorable course for their condition. 

## References

[B1] Guidon AC, Amato AA (2020). COVID-19 and neuromuscular disorders. Neurology.

[B2] Laventhal NT, Graham RJ, Rasmussen SA, Urion DK, Kang PB (2020). Ethical decision-making for children with neuromuscular disorders in the COVID-19 crisis. Neurology.

[B3] Grossman SN, Han SC, Balcer LJ, Kurzweil A, Weinberg H, Galetta SL (2020). Rapid implementation of virtual neurology in response to the COVID-19 pandemic. Neurology.

[B4] Veerapandiyan A, Connolly AM, Finkel RS, Arya K, Mathews KD, Smith EC (2020). Spinal muscular atrophy care in the COVID-19 pandemic era. Muscle Nerve.

[B5] Solé G, Salort-Campana E, Pereon Y, Stojkovic T, Wahbi K, Cintas P (2020). Guidance for the care of neuromuscular patients during the COVID-19 pandemic outbreak from the French Rare Health Care for Neuromuscular Diseases Network. Rev Neurol (Paris).

[B6] Studart-Neto A, Guedes BF, Tuma RLE, Camelo AE, Kubota GT, Iepsen BD (2020). Neurological consultations and diagnoses in a large, dedicated COVID-19 university hospital. Arq Neuropsiquiatr.

[B7] Dowling JJ, D Gonorazky H, Cohn RD, Campbell C (2018). Treating pediatric neuromuscular disorders: the future is now. Am J Med Genet A.

[B8] Korinthenberg R (2017). Neuromuscular disorders in children and adolescents. Neuropediatrics.

[B9] Camelo-Filho AE, Silva AMS, Estephan EP, Zambon AA, Mendonça RH, Souza PVS (2020). Myasthenia Gravis and COVID-19: clinical characteristics and outcomes. Front Neurol.

[B10] Dalakas MC (2020). Guillain-Barré syndrome: The first documented COVID-19-triggered autoimmune neurologic disease: more to come with myositis in the offing. Neurol Neuroimmunol Neuroinflamm.

[B11] Bellino S, Punzo O, Rota MC, Del Manso M, Urdiales AM, Andrianou X (2020). COVID-19 disease severity risk factors for pediatric patients in Italy. Pediatrics.

[B12] Di Stefano V, Battaglia G, Giustino V, Gagliardo A, D'Aleo M, Giannini O (2021). Significant reduction of physical activity in patients with neuromuscular disease during COVID-19 pandemic: the long-term consequences of quarantine. J Neurol.

[B13] Handberg C, Werlauff U, Højberg A-L, Knudsen LF (2021). Impact of the COVID-19 pandemic on biopsychosocial health and quality of life among Danish children and adults with neuromuscular diseases (NMD)-Patient reported outcomes from a national survey. PLoS One.

[B14] Pfefferbaum B, North CS (2020). Mental health and the Covid-19 Pandemic. N Engl J Med.

